# Individual Correlates of Infectivity of Influenza A Virus Infections in Households

**DOI:** 10.1371/journal.pone.0154418

**Published:** 2016-05-06

**Authors:** Tim K. Tsang, Vicky J. Fang, Kwok-Hung Chan, Dennis K. M. Ip, Gabriel M. Leung, J. S. Malik Peiris, Benjamin J. Cowling, Simon Cauchemez

**Affiliations:** 1 WHO Collaborating Centre for Infectious Disease Epidemiology and Control, School of Public Health, Li Ka Shing Faculty of Medicine, The University of Hong Kong, Hong Kong Special Administrative Region, China; 2 Department of Microbiology, Li Ka Shing Faculty of Medicine, The University of Hong Kong, Hong Kong Special Administrative Region, China; 3 Centre of Influenza Research, Li Ka Shing Faculty of Medicine, The University of Hong Kong, Hong Kong Special Administrative Region, China; 4 Mathematical Modelling of Infectious Diseases Unit, Institut Pasteur, Paris, France; Icahn School of Medicine at Mount Sinai, UNITED STATES

## Abstract

**Background:**

Identifying individual correlates of infectivity of influenza virus is important for disease control and prevention. Viral shedding is used as a proxy measure of infectivity in many studies. However, the evidence for this is limited.

**Methods:**

In a detailed study of influenza virus transmission within households in 2008–12, we recruited index cases with confirmed influenza infection from outpatient clinics, and followed up their household contacts for 7–10 days to identify secondary infections. We used individual-based hazard models to characterize the relationship between individual viral shedding and individual infectivity.

**Results:**

We analyzed 386 households with 1147 household contacts. Index cases were separated into 3 groups according to their estimated level of viral shedding at symptom onset. We did not find a statistically significant association of virus shedding with transmission. Index cases in medium and higher viral shedding groups were estimated to have 21% (95% CI: -29%, 113%) and 44% (CI: -16%, 167%) higher infectivity, compared with those in the lower viral shedding group.

**Conclusions:**

Individual viral load measured by RT-PCR in the nose and throat was at most weakly correlated with individual infectivity in households. Other correlates of infectivity should be examined in future studies.

## Introduction

Influenza virus has low to moderate transmissibility in the community. The median effective reproductive numbers generally estimated to 1.28 (inter-quartile range: 1.19 to 1.37) and 1.46 (inter-quartile range: 1.30 to 1.70) for seasonal influenza and 2009 pandemic influenza respectively [1]. The household is an important setting for influenza virus transmission since up to 30% of influenza transmission occurs there [2–4]. Once one household member becomes infected the risk of transmission to other household members is often in the range 10% to 20% [5]. Moreover, studies in households make it possible to identify characteristics associated with increased transmission in confined settings, and observe the full range of illness associated with influenza [4]. Such empirical estimates offer insights into the natural history of influenza, and assist preparedness for influenza epidemics and pandemics by improving the calibration of mathematical models [3,6–8].

One particular interest is the relationship between viral load in the nose and throat of infected individuals and infectivity towards others, since viral load has been used as a proxy measure of infectivity. In a number of studies, infectivity was assumed to be proportional to viral load and the duration of viral shedding was used as a proxy measure of the duration of infectiousness [9–17]. However, their relationship is rarely validated or confirmed in natural settings.

In a previous study [18], the analysis of large prospective studies of transmission of influenza A virus in households in Hong Kong from 2008 to 2012 [13,19] revealed that using average temporal trends in viral loads after illness onset to approximate average temporal trends in infectivity did not explain much of the variability in the timing of secondary infections in households. Here, we explore and quantify the relationship between influenza A viral shedding and infectivity on an individual basis to investigate whether cases with higher viral loads are significantly more infectious than others.

## Methods

### Study subjects

We conducted large community-based studies of the household transmission of influenza virus in Hong Kong [13,19]. In these studies, we recruited outpatients with acute respiratory illness within 2 days after illness onset, who lived in a household with at least 2 other persons none of whom reported recent illness in the preceding 14 days before the time of the first visit. Then they were tested by the QuickVue Influenza A+B test (Quidel, San Diego, CA). We then further followed up participants with a positive result on the rapid test and along with their household contacts, involving 3 home visits over approximately 7 days. During each home visit, nose and throat swab specimens were collected from all subjects and their household contacts regardless of the presence of respiratory symptoms. Daily symptoms for index cases and their household contacts were recorded in symptom diaries for the duration of follow-up. Subjects recruited from January 2008 to June 2009 were part of a randomized controlled trial of enhanced hand hygiene with or without surgical face masks randomly allocated on a household basis [19]; while subjects subsequently recruited in the summer of 2009 and afterwards were part of a comparative study of seasonal and pandemic influenza virus transmission in households and a simple hand hygiene intervention was given to all households [13]. Only households in which index cases had PCR-confirmed influenza A virus infection were included in our analyses.

### Ethical approval

All subjects 18 years of age and older gave written informed consent, and proxy written consent was obtained from parents or legal guardians for children aged 17 years of age or younger, with additional written assent from those 8 to 17 years of age. The study protocol was approved by the Institutional Review Board of the University of Hong Kong.

### Laboratory methods

Paired nasal and throat swabs were pooled immediately after collection in viral transport medium and delivered to the laboratory for cryopreservation at -70°C within 24 hours of collection. The swabs were subsequently tested by quantitative reverse transcription polymerase chain reaction (PCR) to detect influenza A virus and quantify virus shedding. Total nucleic acid was extracted by using the NucliSens easy MAG extraction system (bioMerieux, Boxtel, The Netherlands) according to the manufacturer’s instructions. Twelve microliters of extracted nucleic acid with a random primer was used to prepare complementary DNA by using an Invitrogen Superscript III kit (Invitrogen), as described elsewhere [20]. Detection of influenza A virus was conducted in a PCR assay as previously described [21]. At the end of the assay, PCR products were subjected to a melting-curve analysis to determine the specificity of the assay. The lower limit of detection (LLOD) of the PCR assay was approximately 900 virus gene copies per milliliter.

### Statistical analysis

We defined PCR-confirmed influenza virus infection as a positive result on testing of one or more pooled nasal and throat specimens from household contacts collected during the follow-up period. Illness onset time for PCR-confirmed influenza virus infection was defined as the first day when the subject reported at least 2 of the following 7 signs or symptoms: runny nose, cough, sore throat, headache, phlegm, myalgia and fever [9]. Index cases with PCR-confirmed influenza B virus infection were excluded in the analysis because patients with influenza B virus infection showed different viral shedding pattern [22] and the risk factors for influenza B virus infections could be different compared with influenza A virus infections [23].

To explore the association between viral load and infectivity of individual persons, we used the viral load at symptom onset as a single measure of level of viral shedding of individuals. However, the viral shedding trajectories were only available for some days at or after symptom onset. To address this issue of incomplete observation, we first fitted a log-linear censored regression model [10,24], which accounted for the LLOD of the PCR assay [25] and allowed for separate intercepts for each individual but a common slope for the observed viral shedding data that was supported by other studies [9,12,18]. We then used the fitted model to predict the viral load at symptom onset for each case. After that, we separated cases into three groups with lower, medium and higher viral loads, based on the tertiles of the predicted level of viral shedding at symptom onset, and compared the relative infectivity among these three groups.

We characterized the transmission dynamics in households and the factors affecting infectivity or susceptibility by using an individual based hazard model [18,26,27]. The model described the risk of PCR-confirmed infection among household contacts as depending on the time since symptom onset in any other infected persons in each household. The model allowed for infections from outside the household (“community infections”) and infections via other household contacts rather than the index case (“tertiary infections”).

We then estimated the relative infectivity of these three groups with adjustment for age and receipt of influenza vaccination that might influence individual susceptibility in household contacts. We also incorporated factors that might influence individual infectivity including age, oseltamivir treatment, and subtype differences [13,18,26–29]. We also further explored if the presence of specific symptoms or combinations of symptoms were correlated with individual infectivity.

In the main analyses, we excluded households that included more than one person with symptom onset at recruitment (i.e. multiple index cases) from the analyses according to the study design. As a sensitivity analysis, we repeat our analyses with all households regardless of the presence of multiple index cases.

We conducted statistical analyses in a Bayesian framework. We constructed a Markov Chain Monte Carlo algorithm [30] to fit the transmission model and estimate the parameters. One particular feature of our study design is that there were no household contacts with symptom onset at or before the recruitment day, and we accounted for this by using conditional likelihood in the statistical model [18]. All statistical analyses were conducted using R version 3.0.1 (R Foundation for Statistical Computing, Vienna, Austria) and MATLAB 7.8.0 (MathWorks Inc., Natick, MA). Raw data and R syntax to reproduce our analyses are available via Dryad (http://dx.doi.org/10.5061/dryad.1p3kn).

## Results

From February 2008 through December 2012 we used rapid tests to screen 4553 cases in outpatient clinics, of whom 931 were positive for influenza A or B. Of those, 705 agreed to further follow-up and 453 of them had PCR-confirmed influenza A virus infection. 67 households were excluded from the analysis since they had more than one person with symptom onset at recruitment (i.e. multiple index cases). Households with multiple index cases that were excluded from the main analysis had more children on average than included households. Other characteristics of the included and excluded index cases, including observed viral loads at recruitment, were similar (S1 Table). In total 386 households with an index case with PCR-confirmed influenza A were included in our analysis. The age of the index case varied significantly with the viral shedding groups, while other characteristics of index cases or household contacts were similar (Table 1).

**Table 1:** Characteristics of index cases classified as having lower, medium or higher levels of viral shedding at symptom onset, and their household contacts.

	Level of viral shedding			
Characteristic	Lower	Medium	Higher	p-value
***Index cases***				
**No. of cases**	129	128	129	
**Mean (Range) of Observed viral shedding (log**_**10**_ **copies/mL) at recruitment**				
**Recruited at symptom onset**	6.28 (3.56, 9.04)	7.64 (6.13, 9.04)	8.24 (5.49, 9.41)	<0.001
**Recruited at 1 day after symptom onset**	5.71 (3.42, 7.74)	6.45 (4.01, 8.85)	7.63 (4.87, 9.51)	<0.001
**Recruited at 2 days after symptom onset**	4.69 (2.95, 6.96)	5.97 (3.94, 8.82)	7.10 (4.33, 9.36)	<0.001
**Mean (Range) of predicted viral shedding (log**_**10**_ **copies/mL) at symptom onset**	6.48 (5.72, 6.85)	7.16 (6.85, 7.51)	8.04 (7.51, 9.54)	
**Age**				
**Mean (range)**	15 (1, 81)	23 (1, 72)	18 (0, 79)	0.001
**≤18yr**	105 (81%)	71 (55%)	88 (68%)	
**18–50 yr**	17 (13%)	41 (32%)	36 (28%)	
**>50 yr**	7 (5%)	16 (12%)	5 (4%)	<0.001
**Male sex**	57 (44%)	64 (50%)	71 (55%)	0.218
**Prior vaccination**	26 (20%)	22 (17%)	19 (15%)	0.516
**Oseltamivir treatment**^1^	61 (47%)	52 (41%)	43 (33%)	0.074
**Number of household contacts**				
**2**	41 (32%)	53 (41%)	42 (33%)	
**3**	51 (40%)	44 (34%)	52 (40%)	
**4**	34 (26%)	27 (21%)	25 (19%)	
**5**	2 (2%)	4 (3%)	6 (5%)	
**6**	1 (1%)	0 (0%)	4 (3%)	0.512
**Number of secondary cases in household**				
**0**	103 (80%)	100 (78%)	105 (81%)	
**1**	23 (18%)	25 (20%)	18 (14%)	
**2**	2 (2%)	3 (2%)	5 (4%)	
**3**	1 (1%)	0 (0%)	1 (1%)	0.808
***Household contacts***				
**No. of contacts**	387	366	394	
**Age**				
**Mean (range)**	32 (1, 90)	32 (0, 92)	31 (0, 97)	0.657
**≤18yr**	69 (18%)	79 (22%)	79 (20%)	
**18–50 yr**	246 (64%)	220 (60%)	252 (64%)	
**>50 yr**	72 (19%)	67 (18%)	63 (16%)	0.591
**Male sex**	154 (40%)	130 (36%)	150 (38%)	0.478
**Prior vaccination**	44 (12%)	44 (12%)	62 (16%)	0.167
**Number of Secondary infection (Secondary infection risk)**				
**Overall**	30 (8%)	31 (8%)	31 (8%)	0.928
**≤18yr**	13 (19%)	8 (10%)	6 (8%)	0.090
**18–50 yr**	15 (6%)	21 (10%)	20 (8%)	0.381
**>50 yr**	2 (3%)	2 (3%)	5 (8%)	0.271

^1^only oseltamivir treatment started within 48 hours after onset was classified as treatment group

**Fig 1:**
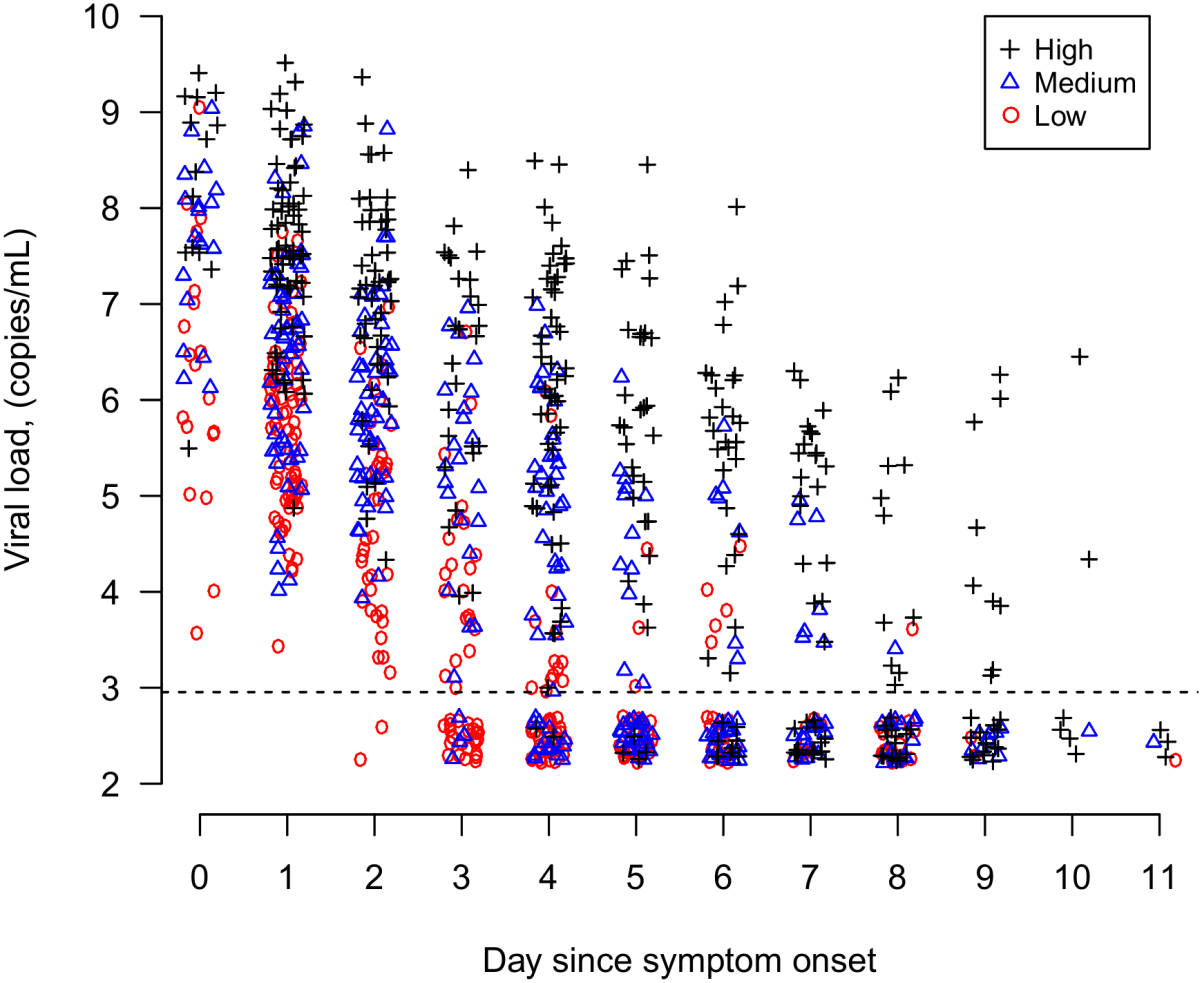
Viral shedding pattern from observed data for the high, medium or low viral shedding groups.

Three viral shedding groups were constructed based on the tertiles of the predicted viral load at symptom onset from the random effects log-linear censored regression model that was fitted to all available data on viral loads. The observed viral shedding patterns for the three viral shedding groups are shown in Fig 1. As expected, index cases classified in the higher viral shedding group had generally higher observed viral loads than those in the medium viral shedding group and those in the medium shedding group had observed higher viral loads than those in the lower viral shedding group over time since onset, with some overlap between groups (S2 Table). The overall secondary infection risk among household contacts in the studies was 8%. The overall and age-specific secondary infection risks were similar among the household contacts of each of the three groups of index cases with higher, medium, or lower levels of viral shedding (Table 1).

The results of the individual based hazard model are shown in Table 2. It is estimated that index cases in the high and medium shedding groups were estimated to have 21% (95% CI: -29%, 113%) and 44% (CI: -16%, 167%) higher infectivity, compared with those in the lower viral shedding group. The association was therefore not statistically significant. Children (≤ 18 yr) were associated with 65% (CI: -1%, 183%) higher infectivity, compared with adults (>18 yr). We also found that children (≤ 18 yr) household contacts were more susceptible than middle-aged adult contacts (19–50 yr) (relative susceptibility: 1.78; 95% CI: 1.08 to 2.73). In a sensitivity analysis, the estimates from the model were similar when households with multiple index cases were added to the analysis (S3 Table).

**Table 2:** Factors affecting influenza susceptibility and infectivity in the household transmission model.

Characteristics	Adjusted risk ratio
***Factors affecting infectivity***	
**Age ≤18yr vs >18 yr (Ref)**	1.65 (0.99, 2.83)
**Oseltamivir treatment**^1^	0.88 (0.52, 1.42)
**Level of viral shedding at symptom onset:**	
** Medium vs Low (Ref)**	1.21 (0.71, 2.13)
** High vs Low (Ref)**	1.44 (0.84, 2.67)
**Subtype:**	
** sH3N2 vs sH1N1 (Ref)**	1.52 (0.97, 2.50)
** pH1N1 vs sH1N1 (Ref)**	1.13 (0.49, 2.38)
** A(Unsubtypable) vs sH1N1 (Ref)**	0.24 (0.09, 0.56)
***Factors affecting susceptibility***	
**Age ≤18 vs 19–50 yr (Ref)**	1.78 (1.08, 2.73)
**Age >50 vs 19–50 yr (Ref)**	0.71 (0.34, 1.34)
**Vaccination**	0.93 (0.47, 1.63)

^1^only oseltamivir treatment started within 48 hours after onset was classified as treatment group

We further explored if individual symptoms could affect infectivity by including them into the main model one at a time, and found that index case with fever was associated with 94% (CI: 9%, 330%) higher infectivity, compared with those without fever. We also found that index cases with influenza-like illness (ILI), defined by the presence of fever with either sore throat or cough, was associated with 84% (CI: 11%, 256%) higher infectivity, compared with those without fever. No other symptoms had a statistically significant association with infectivity (Table 3).

**Table 3:** Association between symptoms and infectivity.

Symptom^1^	Adjusted risk ratio
**Fever**	**1.94 (1.09, 4.30)**
**Sore throat**	0.77 (0.47, 1.27)
**Cough**	1.07 (0.52, 2.66)
**Runny nose**	0.92 (0.42, 4.57)
**Phlegm**	0.93 (0.55, 1.84)
**Muscle pain**	1.00 (0.63, 1.59)
**Headache**	1.04 (0.64, 1.64)
**Any 3 or more of the above signs/symptoms**	1.24 (0.49, 3.82)
**ILI defined as fever plus cough or sore throat**	**1.84 (1.11, 3.56)**

^1^Factors in the table were added one at a time to the main model shown in Table 2 and the effect of that factor was estimated in each model.

## Discussion

In a previous study [18], we investigated whether viral shedding trajectories could predict the timing of secondary infections in households. We found that assuming infectivity is proportional to the average trajectory of shedding led to underestimating the proportion of secondary infections occurring within the first 3 days after symptom onset in index cases in households. Here, we further explored the relationship between individual viral shedding, measured by PCR on pooled nose and throat swabs, and individual infectivity in influenza virus transmission in household settings. We divided index cases into three groups with low, medium and high level of viral shedding based on their predicted viral loads at symptom onset, and found no statistically significant differences in the infectivity of the different groups (Table 2). However, given that the point estimates of the relative infectivity of medium and higher viral shedding groups, compared with lower viral shedding group, were 21% and 44% respectively without statistical significance. The direction of effects is consistent with a dose-response relationship, and the effect sizes could have clinical and public health significance. This indicates that our study was underpowered to detect smaller associations of viral load with infectivity. In our analysis we found that younger index cases and presence of fever were associated with higher infectivity, suggesting that these factors may be stronger correlates of infectivity.

Whereas it has commonly been assumed that persons with higher viral load would be more infectious to others around them, our study suggested that there was at most a weak association between viral load and infectivity. One potential explanation for the lack of stronger association is that viral shedding measured by PCR in the nose and throat does not fully capture the amount of infectious virus excreted into the environment by an infected person. For example, a recent study found only a weak correlation (r = 0.29) between influenza virus concentration in aerosols in exhaled breath and viral load in nasopharyngeal swabs from the same subjects [31]. This indicates that viral load measured by PCR in the nose and throat may be a weak correlate of infectivity. Therefore, we should examine other measures of shedding that might better correlate with infectivity, such as measurement of viral shedding in exhaled breath.

Another potential explanation for this finding is that infected persons with low viral loads are still highly infectious in households because of intense and frequent contact in this setting. While the lack of a correlation between index case viral load and infectivity could be explained if most secondary infections had been infected in the community, our analysis of the homology in virus sequences between infections in index cases and household contacts confirmed that almost all infections in household contacts were acquired within the household [32].

Nose and throat swabs were pooled in our studies and this may have had an impact on our results. Pooling the nose and throat samples may lower the viral loads and therefore may decrease accuracy to measure the viral loads in an infected person. However, we might expect a high correlation in viral loads in the two collection sites in the upper respiratory tract. It is also possible that by pooling the nose and throat swabs, we have masked the significance of viral load in one specific site. The lower limit of detection of the PCR assay in our study was 900 virus gene copies per milliliter and therefore some secondary infections with viral loads lower than this limit may have been missed. If the viral shedding level of index cases is associated with that in their corresponding secondary index cases, there could be bias in our results. In future iterations of the work, it will be interesting to test a range of sites (including viral load in nose, throat, and exhaled breath) to determine if any one of these could separately correlate with individual infectivity.

We explored the association between infectivity and presence of symptoms and found that fever was associated with higher infectivity, which was also reported by another household transmission study [33]. Two other household transmission studies did not detect an association between fever and infectivity, perhaps because of lack of power [34], or because secondary infections were not laboratory confirmed [26]. If fever were a proxy of high infectivity for some biological reason, it could potentially be used as an indicator of cases particularly worthy of isolation or sequestration in a pandemic or severe epidemic. On the other hand, higher infectivity of febrile cases could occur because a febrile case required closer care from close contact. In our analysis the statistically significant association between fever and infectivity was adjusted for viral shedding tertiles. Therefore, we believe that the association between fever and higher infectivity is not confound by viral shedding. Nevertheless, fever may have interacted with the role of viral load, and we did not have sufficient sample size to examine potential interactions between signs/symptoms and viral load. In our study we did not detect a significant association between infectivity and other symptoms (Table 3). Cough was associated with higher infectivity in household transmission studies, but in those particular studies the secondary cases were identified based on clinical symptoms but were not laboratory confirmed [35–37].

Our study has a number of limitations. First, symptomatic index cases were recruited from outpatient clinics. Therefore they had an illness that warranted medical attention and a positive result on a rapid influenza test [38]. Therefore, they might have higher levels of virus shedding than typical influenza cases and hence the generalizability of our results may be limited. Second, since index cases were recruited after symptom onset, pre-symptomatic infectivity was not considered in our study because of lack of information on pre-symptomatic virus shedding. Finally, our study is observational and hence we controlled some confounders such as age and vaccination in our transmission model. However, we cannot rule out the risk of other unidentified confounders.

In conclusion, we found that individual viral shedding measured by RT-PCR in the nose and throat was at most a weak correlate of individual infectivity in household settings, potentially because of a weak correlation between influenza virus concentration in aerosols in exhaled breath and viral load in nose and throat swabs [31], or because transmission in households is so intense that infected persons with low viral loads can still easily infect those around them. Individuals with febrile illness were more infectious to their household contacts perhaps because of greater or more intense contact at home. Other correlates of infectivity should be examined in future studies.

## Supporting Information

S1 Table Characteristics of index cases without or with at least one household contact with symptoms. (DOCX)

S2 Table Observed viral shedding of index cases with low, medium or high level of viral shedding at symptom onset. (DOCX)

S3 Table Factors affecting influenza susceptibility and infectivity in the household transmission model included household with multiple index cases. (DOCX)
