# Supplementary material for: Individual Correlates of Infectivity of Influenza A Virus Infections in Households
Source: PLoS One. 2016 May 6;11(5):e0154418. doi: 10.1371/journal.pone.0154418 (PMC4859516; doi:10.1371/journal.pone.0154418)
Supplement: S1 Table — (DOCX) [file pone.0154418.s001.docx]

**SUPPORTING INFORMATION**

**S1 Table.** Characteristics of index cases without or with at least one household contact with symptoms

| Characteristic | Index cases without household contacts with symptoms | Index cases with at least one household contact with symptoms | p-value |
| --- | --- | --- | --- |
| No. of cases | 386 | 67 |  |
| Mean (Range) of Observed viral shedding (log_10_ copies/mL) at: |  |  |  |
| symptom onset | 7.25 (3.56, 9.41) | 7.14 (4.73, 9.49) | 0.805 |
| 1 day after symptom onset | 6.51 (3.42, 9.51) | 6.83 (3.17, 9.62) | 0.109 |
| 2 days after symptom onset | 5.98 (2.95, 9.36) | 5.8 (2.95, 8.05) | 0.429 |
|  |  |  |  |
| Age |  |  |  |
| Mean (range) | 19 (0, 81) | 13 (2, 53) | 0.010 |
| ≤18yr | 264 (68%) | 55 (82%) |  |
| 18-50 yr | 94 (24%) | 11 (16%) |  |
| >50 yr | 28 (7%) | 1 (1%) | 0.049 |
|  |  |  |  |
| Male sex | 192 (50%) | 38 (57%) | 0.357 |
|  |  |  |  |
| Prior vaccination | 67 (17%) | 9 (13%) | 0.532 |
|  |  |  |  |
| Oseltamivir treatment^1^ | 156 (40%) | 20 (30%) | 0.133 |
|  |  |  |  |
| Number of household contacts |  |  |  |
| 2 | 136 (35%) | 12 (18%) |  |
| 3 | 147 (38%) | 25 (37%) |  |
| 4 | 86 (22%) | 18 (27%) |  |
| 5 | 12 (3%) | 11 (16%) |  |
| 6 | 5 (1%) | 1 (1%) | 0.003 |
|  |  |  |  |
| Number of secondary cases in household^2^ |  |  |  |
| 0 | 310 (80%) | 48 (72%) |  |
| 1 | 67 (17%) | 15 (22%) |  |
| 2 | 7 (2%) | 4 (6%) |  |
| 3 | 2 (1%) | 0 (0%) | 0.148 |

^1^only oseltamivir treatment started within 48 hours after onset was classified as treatment group

^2^Excluded household contacts with presence of symptoms at recruitment
