# Supplementary material for: Individual Correlates of Infectivity of Influenza A Virus Infections in Households
Source: PLoS One. 2016 May 6;11(5):e0154418. doi: 10.1371/journal.pone.0154418 (PMC4859516; doi:10.1371/journal.pone.0154418)
Supplement: S2 Table — (DOCX) [file pone.0154418.s002.docx]

**SUPPORTING INFORMATION**

**S2 Table.** Observed viral shedding of index cases with low, medium or high level of viral shedding at symptom onset

|  | Low | | Medium | | High | |  |
| --- | --- | --- | --- | --- | --- | --- | --- |
| Days since symptom onset | Number of sample below detection limit | Mean of viral load (range) | Number of sample below detection limit | Mean of viral load (range) | Number of sample below detection limit | Mean of viral load (range) | p-value |
| 0 | 0/19 (0%) | 6.28 (3.56, 9.04) | 0/19 (0%) | 7.64 (6.13, 9.04) | 0/14 (0%) | 8.24 (5.49, 9.41) | <0.001 |
| 1 | 0/72 (0%) | 5.71 (3.42, 7.74) | 0/58 (0%) | 6.45 (4.01, 8.85) | 0/64 (0%) | 7.63 (4.87, 9.51) | <0.001 |
| 2 | 2/38 (5%) | 4.79 (3.16, 6.96) | 0/50 (0%) | 5.97 (3.94, 8.82) | 0/47 (0%) | 7.1 (4.33, 9.36) | <0.001 |
| 3 | 25/46 (54%) | 4.27 (2.99, 6.7) | 4/26 (15%) | 5.28 (3.11, 6.96) | 0/27 (0%) | 6.37 (3.96, 8.4) | <0.001 |
| 4 | 31/44 (70%) | 3.7 (2.96, 6.09) | 16/49 (33%) | 5.09 (2.96, 6.98) | 2/52 (4%) | 6.14 (3, 8.49) | <0.001 |
| 5 | 25/28 (89%) | 3.69 (3.01, 4.44) | 25/37 (68%) | 4.59 (3.05, 6.24) | 4/33 (12%) | 5.88 (3.63, 8.45) | <0.001 |
| 6 | 17/22 (77%) | 3.88 (3.47, 4.47) | 24/31 (77%) | 4.6 (3.3, 5.72) | 9/36 (25%) | 5.56 (3.15, 8.01) | <0.001 |
| 7 | 7/7 (100%) | NA | 16/23 (70%) | 4.13 (3.47, 4.96) | 15/35 (43%) | 5.13 (3.48, 6.3) | 0.022 |
| 8 | 14/15 (93%) | 3.61 (3.61, 3.61) | 15/16 (94%) | 3.4 (3.4, 3.4) | 16/27 (59%) | 4.5 (3.03, 6.23) | 0.016 |
| 9 | 2/2 (100%) | NA | 9/9 (100%) | NA | 14/23 (61%) | 4.54 (3.13, 6.26) | NA |
| 10 | 0 | NA | 1/1 (100%) | NA | 4/6 (67%) | 5.39 (4.34, 6.45) | NA |
| 11 | 1/1 (100%) | NA | 1/1 (100%) | NA | 3/3 (100%) | NA | NA |
