# Supplementary material for: Individual Correlates of Infectivity of Influenza A Virus Infections in Households
Source: PLoS One. 2016 May 6;11(5):e0154418. doi: 10.1371/journal.pone.0154418 (PMC4859516; doi:10.1371/journal.pone.0154418)
Supplement: S3 Table — (DOCX) [file pone.0154418.s003.docx]

**SUPPORTING INFORMATION**

**S3 Table.** Factors affecting influenza susceptibility and infectivity in the household transmission model included household with multiple index cases.

| Characteristics | Adjusted risk ratio |
| --- | --- |
| *Factors affecting infectivity* |  |
| Age ≤18yr vs >18 yr (Ref) | 1.70 (1.17, 2.51) |
|  |  |
| Oseltamivir treatment^1^ | 0.76 (0.52, 1.08) |
|  |  |
| Level of viral shedding at symptom onset: |  |
| Medium vs Low (Ref) | 1.45 (0.95, 2.14) |
| High vs Low (Ref) | 1.38 (0.89, 2.12) |
|  |  |
| Subtype: |  |
| sH3N2 vs sH1N1 (Ref) | 1.08 (0.78, 1.54) |
| pH1N1 vs sH1N1 (Ref) | 0.70 (0.36, 1.21) |
| A(Unsubtypable) vs sH1N1 (Ref) | 0.39 (0.23, 0.65) |
|  |  |
| *Factors affecting susceptibility* |  |
| Age ≤18 vs 19-50 yr (Ref) | 2.80 (2.06, 3.80) |
| Age >50 vs 19-50 yr (Ref) | 0.55 (0.30, 0.99) |
|  |  |
| Vaccination | 0.96 (0.60, 1.43) |

^1^only oseltamivir treatment started within 48 hours after onset was classified as treatment group
